# Piperine Inhibits the Activities of Platelet Cytosolic Phospholipase A_2_ and Thromboxane A_2_ Synthase without Affecting Cyclooxygenase-1 Activity: Different Mechanisms of Action Are Involved in the Inhibition of Platelet Aggregation and Macrophage Inflammatory Response

**DOI:** 10.3390/nu6083336

**Published:** 2014-08-22

**Authors:** Dong Ju Son, Satoshi Akiba, Jin Tae Hong, Yeo Pyo Yun, Seock Yeon Hwang, Young Hyun Park, Sung Eun Lee

**Affiliations:** 1 School of Applied Biosciences, Kyungpook National University, Daegu 702-701, Korea; E-Mail: sondj1@hotmail.com; 2 Department of Pathological Biochemistry, Kyoto Pharmaceutical University, Kyoto 607-8414, Japan; E-Mail: akiba@mb.kyoto-phu.ac.jp; 3 College of Pharmacy and Center for Innovative Cancer Therapeutics, Chungbuk National University, Cheongju 361-763, Korea; E-Mails: jinthong@chungbuk.ac.kr (J.T.H.); ypyun@chungbuk.ac.kr (Y.P.Y.); 4 Department of Biomedical Laboratory Science, College of Natural Science, Daejeon 300-716, Korea; E-Mail: syhwang@dju.ac.kr; 5 Department of Food Science and Nutrition, College of Natural Sciences, Soonchunhayng University, Asan 336-745, Korea

**Keywords:** pepper, piperine, platelet aggregation, arachidonic acid, cyclooxygenase, phospholipase A_2_, thromboxane A_2_ synthase, prostaglandins

## Abstract

PURPOSE: Piperine, a major alkaloid of black pepper (*Piper nigrum*) and long pepper (*Piper longum*), was shown to have anti-inflammatory activity through the suppression of cyclooxygenase (COX)-2 gene expression and enzyme activity. It is also reported to exhibit anti-platelet activity, but the mechanism underlying this action remains unknown. In this study, we investigated a putative anti-platelet aggregation mechanism involving arachidonic acid (AA) metabolism and how this compares with the mechanism by which it inhibits macrophage inflammatory responses; METHODS: Rabbit platelets and murine macrophage RAW264.7 cells were treated with piperine, and the effect of piperine on the activity of AA-metabolizing enzymes, including cytosolic phospholipase A_2_ (cPLA_2_), COX-1, COX-2, and thromboxane A_2_ (TXA_2_) synthase, as well as its effect on AA liberation from the plasma membrane components, were assessed using isotopic labeling methods and enzyme immunoassay kit; RESULTS: Piperine significantly suppressed AA liberation by attenuating cPLA_2_ activity in collagen-stimulated platelets. It also significantly inhibited the activity of TXA_2_ synthase, but not of COX-1, in platelets. These results suggest that piperine inhibits platelet aggregation by attenuating cPLA_2_ and TXA_2_ synthase activities, rather than through the inhibition of COX-1 activity. On the other hand, piperine significantly inhibited lipopolysaccharide-induced generation of prostaglandin (PG)E_2_ and PGD_2_ in RAW264.7 cells by suppressing the activity of COX-2, without effect on cPLA_2_; CONCLUSION: Our findings indicate that piperine inhibits platelet aggregation and macrophage inflammatory response by different mechanisms.

## 1. Introduction

Platelet aggregation is a complex, rapidly progressing phenomenon that culminates in the formation of hemostatic plugs and arterial thrombi, which are recognized as potential sources of thromboembolic complications manifesting as atherosclerosis, heart attack, stroke, and peripheral vascular disease. Platelets are activated by various agonists, such as collagen and platelet-activating factor, and undergo a cascade of events that results in the enzymatic metabolism of arachidonic acid (AA) [1,2,3]. AA is derived from the plasma membrane by the action of phospholipase A_2_ (PLA_2_), and undergoes further metabolism by cyclooxygenases (COX) and TXA_2_ synthase to form eicosanoid products such as prostaglandins (PGs), thromboxanes (TX), and other oxygenated derivatives [3,4]. Similarly, activated macrophages participate in the inflammatory response by, among other roles, producing eicosanoid pro-inflammatory mediators through the stimulation of the same AA metabolic cascade that controls the platelet aggregation. Therefore, the modulation of eicosanoid mediators by targeting the enzymes involved in AA metabolism is regarded as a promising therapeutic approach for the treatment of thrombosis and chronic inflammatory diseases [5,6,7,8].

Piperine is a primary alkaloid constituent in black and long pepper (*Piper nigrum* and *Piper longum*, respectively), and has been shown to exhibit diverse biological actions, including anti-cancer, anti-angiogenesis, anti-oxidant, and anti-degenerative properties, as well as to enhance drug bioavailability [9,10,11,12,13,14,15]. Recent studies have demonstrated that piperine also possesses anti-inflammatory properties, elicited by the inhibition of PGE_2_ generation through suppression of COX-2 gene transcription and protein expression [16,17,18,19,20,21,22]. With regard to putative anti-platelet activity, piperine and piperine-enriched ethanol extract of *Piper longum* L. have been reported to inhibit platelet aggregation *in vitro* [23,24,25], but the underlying mechanism is currently poorly understood.

In this study, we investigated a putative anti-platelet aggregation mechanism involving AA metabolism by assessing the effect of piperine on the activity of AA-metabolizing enzymes, including cPLA_2_, COX-1 (an isoform of COX-2), and TXA_2_ synthase, as well as its effect on AA liberation from the plasma membrane components. Furthermore, we evaluated the differences in the inhibitory action of piperine on the activities of AA-metabolizing enzymes in platelet aggregation and macrophage inflammatory responses.

## 2. Materials and Methods

### 2.1. Materials

Piperine was obtained from Sigma-Aldrich (St. Louis, MO, USA). Collagen and AA were purchased from Chrono-Log Co. (Havertown, PA, USA). TXB_2_, PGD_2_, PGE_2_, and methyl-arachidonyl-fluorophosphonate (MAFP) were purchased from Cayman Chemical Co. (Ann Arbor, MI, USA). [^3^H]AA (100 Ci/mmol) and 1-stearoyl-2-[^3^H]arachidonoyl-*sn*-glycero-3-phosphocholine ([^3^H]SAPC, 172 Ci/mmol) were purchased from PerkinElmer, Inc. (Waltham, MA, USA). Cell culture materials were purchased from Gibco-BRL (Rockville, MD, USA). Bacterial lipopolysaccharide (LPS) from *Escherichia coli*, NS-398 [*N*-(2-cyclohexyloxy-4-nitrophenyl) methanesulfonamide], indomethacin, and imidazole were obtained from Sigma-Aldrich.

### 2.2. Preparation of Platelets

Two-month-old male New Zealand white rabbits were purchased from Samtako Bio Korea Inc. (Osan, Korea) and acclimated for 1 week at a temperature of 24 ± 11 °C and a humidity of 55% ± 5%. The animals had free access to a standard rabbit pellet diet and drinking water before experiments. Fresh blood was collected from the ear artery of New Zealand White rabbits and the anti-coagulant 1% EDTA was added in the ratio 1:9 (v/v, anti-coagulant/whole blood). Platelet-rich plasma (PRP) was obtained by centrifugation at 230× *g* for 10 min at room temperature. The platelets separated from the PRP were washed twice with HEPES buffer (137 mM NaCl, 2.7 mM KCl, 1 mM MgCl_2_, 5.6 mM glucose, 3.8 mM HEPES, 0.4 mM ethylene glycol tetraacetic acid [EGTA], 0.35% bovine serum albumin [BSA], pH 6.5), as described previously [26]. The platelets were counted by Coulter counter (Beckman Coulter Inc., Brea, CA, USA) and adjusted to the cell concentration of 3 × 10^8^ platelets/mL in HEPES buffer (pH 7.4) for subsequent experiments. All animal studies were carried out at Soonchunhyang University. This study was conducted in accordance with the ethical guideline of the Soonchunhyang University Institutional Animal Care and Use Committee.

### 2.3. Cell Culture

RAW264.7 cells, obtained from American Type Culture Collection (Manassas, VA, USA), were cultured in Dulbecco’s modified eagle medium (DMEM, Gibco-BRL) supplemented with 10% heat-inactivated fetal bovine serum (FBS), 100 U/mL of penicillin, and 100 μg/mL streptomycin at 37 °C under humidified air containing 5% CO_2_ inside a CO_2_ incubator. Cells were plated in 35-mm culture dishes at 6 × 10^5^ cells for the following experiments.

### 2.4. Platelet Aggregation Assay

Platelet aggregation was measured using the turbidimetric method with a four-channel aggregometer (470-vs, Chrono-log Co.) as described previously [26]. Briefly, platelets were incubated at 37 °C for 3 min in the aggregometer with piperine at a range of concentrations (100, 200, and 300 μM) in the presence of 1 mM CaCl_2_. Platelet aggregation was induced by the sequential addition of collagen (1 μg/mL), AA (100 μM), and U46619 (1 μM). The maximal platelet aggregation rate was recorded over 10 min with continuous stirring. The percentage of platelet aggregation (% of vehicle-treated control) following incubation with each inducing agent was calculated by the following formula: (*X_max_ piperine-treated ×* 100)/*X_max_ vehicle-treated*, where *X_max_* is the maximum aggregation rate of vehicle- or piperine-treated platelets in each aggregation assay. IC_50_ values (inhibition of 50% of the aggregation) were determined from the concentration-response curves of logarithmic plots of test substance concentration *vs.* % inhibition of aggregation).

### 2.5. Measurement of Arachidonic acid Liberation

For the measurement of AA liberation, isotopic labeling methods for platelets and macrophages were used as described previously [26,27]. Briefly, PRP or RAW264.7 cells were incubated with [^3^H]AA (1 μCi/mL) at 37 °C for 1.5 h or 24 h, respectively. Following incubation, labeled platelets were washed with HEPES buffer, while RAW264.7 cells were washed with PBS containing 0.01% BSA. In order to assess the effect of piperine on AA liberation, labeled platelets were treated with 100, 200, and 300 μM piperine for 3 min in HEPES buffer containing 1 mM CaCl_2_, while RAW264.7 cells were treated with 10, 50, and 10 μM piperine for 24 h with DMEM containing 0.01% BSA in the presence of 100 μM BW755C (3-amino-1-[m-(trifluoromethyl) phenyl]-2-pyrozoline, an inhibitor of both COX and lipoxygenase [28]). The platelets were treated with collagen (10 μg/mL) for 10 min, while RAW264.7 cells were incubated with LPS (1 μg/mL) for 12 h to stimulate AA liberation. The reaction was terminated by the addition of ice-cold chloroform/methanol/HCl (200:200:1, v/v/v). Lipids were extracted and separated by thin-layer chromatography (TLC) on Silica Gel G plates using petroleum ether/diethyl ether/acetic acid (40:40:1, v/v/v) as the developing system. The area corresponding to free fatty acids and other lipids (diacylglycerol, tricylglycerol, and phospholipids) was scraped off the TLC plate, and the radioactivity of each fraction was determined by liquid scintillation counting. The radioactivity signal corresponding to the liberated [^3^H]AA was corrected by adjusting the total radioactivity.

### 2.6. cPLA_2_ Activity Assay

For the cPLA_2_ assay, the PRP and RAW264.7 cells were treated with a range of concentrations of piperine as described above. To assess the effect of piperine on cPLA_2_ activity in collagen-stimulated platelets, piperine-pretreated PRP was stimulated by incubation with collagen (10 μg/mL) for 1.5 h, and the mixture was centrifuged and lysed as described by Hashizume *et al.* [29]. The lysates were subsequently centrifuged at 100,000× *g* at 4 °C for 1 h, and the cPLA_2_ activity in the resultant supernatant (cytosol fraction) was determined as previously described [29]. Briefly, the supernatant was incubated with a mixture of [^3^H]SAPC and unlabeled SAPC (250 Ci/mol, 2 μM) at 37 °C for 15 min in the presence of 5 mM dithiothreitol, a secretory PLA_2_ inhibitor. After lipid extraction, liberated [^3^H]AA was analyzed as described above, and the enzyme activity was calculated.

To assess the effect of piperine on cPLA_2_ activity in LPS-stimulated macrophages, piperine-pretreated RAW264.7 cells were stimulated by 1 μg/mL for 1 h, and the cells were collected and lysed as described previously [30]. The lysates were centrifuged and the cPLA_2_ activity in the resultant supernatant was determined as described above.

### 2.7. Measurement of Prostaglandin Generation

RAW264.7 cells were cultured for 24 h, and then labeled with [^3^H]AA (1 μCi/mL) at 37 °C for 24 h as described above. In order to assess the effect of piperine on PGs generation in LPS-stimulated RAW264.7 cells, labeled cells were treated with piperine at a range of concentrations (10, 50, and 100 μM) for 24 h, and then stimulated by 1 μg/mL LPS for 12 h. Lipids in the medium and cells were extracted and separated by TLC using an upper phase of ethyl acetate/isooctane/acetic acid/water (9:5:2:10, v/v/v/v) as the developing system. The area corresponding to each PG was scraped off, and the radioactivity of each fraction determined by liquid scintillation counting.

### 2.8. COX Activity Assay

We measured the conversion of exogenous AA to TXB_2_, PGE_2_, and PGD_2_ as an index of COX activity as described previously [6,26,31,32]. In order to assess the effect of piperine on platelet COX-1 activity, PRP was treated with piperine at a range of concentrations (100, 200, and 300 μM) for 3 min in the presence of 1 mM CaCl_2_, and subsequently incubated with a mixture of [^3^H]AA (1 μCi/mL) and unlabeled AA (2 μM) for 10 min. The reaction was terminated by the addition of a stop solution (2.6 mM EGTA containing 130 μM BW755C). Lipids were extracted and the radioactivity corresponding to [^3^H]TXB_2_ and [^3^H]PGD_2_ was measured as described previously [26]. To assess the effect of piperine on macrophage COX-2 activity, RAW264.7 cells were treated with piperine at a range of concentrations (10, 50, and 100 μM) for 24 h. The cells were stimulated with LPS in the presence of a mixture of [^3^H]AA (1 μCi/mL) and unlabeled AA (2 μM) for 1 h. Following the extraction of lipids from the medium and cells, the radioactivity of [^3^H]PGE_2_ and [^3^H]PGD_2_ were determined as described above.

### 2.9. TXA_2_ Synthase Activity Assay

In platelets, TXA_2_ synthase catalyzes the conversion of PGH_2_ to TXA_2_, a potent inducer of aggregation. Because TXA_2_ is highly unstable, we measured the concentration of the stable metabolite TXB_2_ formed from PGH_2_ in rabbit platelets as described previously [26]. In order to assess the effect of piperine on platelet TXA_2_ synthase activity, PRP was treated with piperine at a range of concentrations (100, 200, and 300 μM) for 3 min in the presence of 50 μM COX inhibitor indomethacin in an aggregometer cuvette, and subsequently incubated with 5 μM PGH_2_ for 3 min. The reaction was terminated by the addition of a 2 mM EGTA stop solution containing 0.1 M KCl and placing the mixture on ice. The content of the cuvette was transferred to an Eppendorf tube and centrifuged at 13,000× *g* at 4 °C for 4 min. TXB_2_ concentration in the supernatant was measured using an enzyme immunoassay kit (Amersham Pharmacia Biotech Inc., Buckinghamshire, UK).

### 2.10. Statistical Analysis

Data are as means ± standard deviation (S.D.) of the indicated number of experiments. Student’s *t*-test was used for statistical analysis. A *p* value less than 0.05 was considered statistically significant.

## 3. Results

### 3.1. Piperine Inhibited Platelet Aggregation Induced by Collagen and AA, but Not U46619

**Figure 1:**
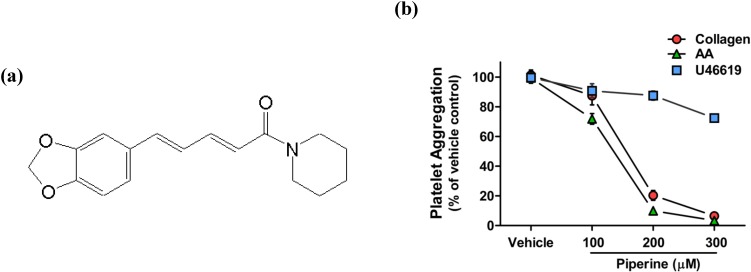
Piperine inhibited the induction of platelet aggregation by collagen and arachidonic acid, but not U46619. (**a**) Chemical structure of piperine; (**b**) Piperine-dependent inhibition of platelet aggregation. Washed platelets (3 × 10^8^ platelets/mL) were pre-incubated with piperine for 3 min in the presence of 1 mM CaCl_2_ and stimulated with 1 μg/mL collagen (red circles), 100 μM AA (blue squares), or 1 μM U46619 (green triangles) in an aggregometer. The change in light transmission of the platelet suspension following 10 min of stimulation with each agonist was normalized to the light transmission of suspensions treated without piperine (taken as 100%). Each point represents the mean ± S.D. of three separate experiments.

We have previously reported that piperine isolated from *Piper longum* L. exerts anti-platelet activity [24]. To investigate a possible mechanism of action, the current study investigated the anti-platelet activity of this new piperine compound. Piperine (Figure 1a) inhibited collagen- and AA-induced platelet aggregation in a concentration-dependent manner, with IC_50_ values of 158.0 and 134.2 μM, respectively (Figure 1b). However, piperine showed only a mild inhibitory effect on platelet aggregation induced by TXA_2_ receptor agonist U46619 [33] (IC_50_ > 300.0 μM) (Figure 1b).

### 3.2. Piperine Inhibited Prostaglandin Generation in Lipopolysaccharide-Stimulated RAW264.7 Cells

**Figure 2:**
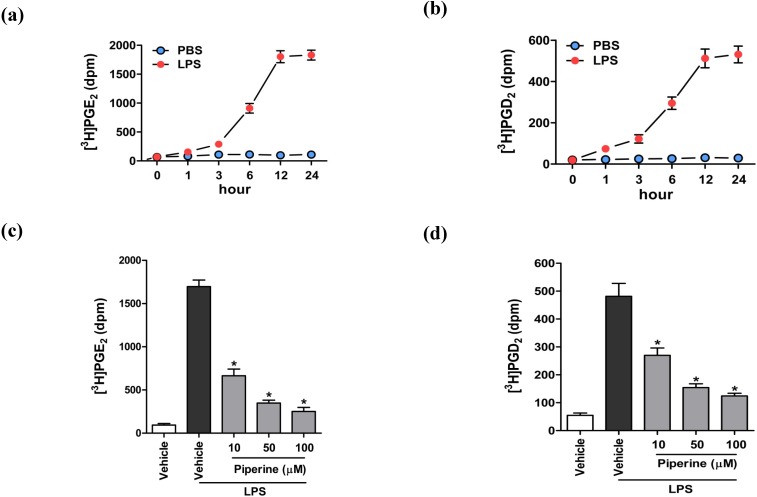
Piperine inhibited the generation of PGE_2_ and PGD_2_ in LPS-stimulated RAW264.7 cells. [^3^H]AA-labeled cells were treated with 1 μg/mL LPS (red circles) or PBS (blue circles), and an evaluation of the time-course of LPS-induced generation of PGE_2_ (**a**) and PGD_2_ (**b**) was performed by quantifying the radioactivity signal of metabolites. [^3^H]AA-labeled cells were treated with piperine (gray bars) or vehicle (DMSO, white and black bars) for 24 h, and stimulated with 1 μg/mL LPS for 12 h. We measured the radioactivity signal corresponding to [^3^H]PGE_2_ (**c**) and [^3^H]PGD_2_; (**d**). Data are expressed as means ± S.D. of three separate experiments. * *p* < 0.05 *vs.* vehicle controls (black bars).

PGs are as one of the major inflammatory mediators [4,5,34,35]. Therefore, we measured the generation of PGs, focusing on PGE_2_ and PGD_2_, in murine macrophages as a marker of pro-inflammatory processes. In our efforts to identify the optimal conditions for the induction of PGs generation in [^3^H]AA-labeled RAW264.7 cells, a time-course study showed that maximal generation of [^3^H]PGE_2_ and [^3^H]PGD_2_ occurred at 12 h post LPS stimulation, whereas PBS as a vehicle control was found to elicit no changes on the basal level of PGs (Figure 2a,b). In subsequent studies, we investigated the effect of piperine on PGs generation in LPS-stimulated RAW264.7 cells under experimental conditions determined to be optimal, *i.e.*, stimulation with 1 μg/mL LPS for 12 h. Piperine significantly inhibited the generation of both PGE_2_ (Figure 2c) and PGD_2_ (Figure 2d) in a concentration-dependent manner, with IC_50_ values of 7.7 and 10.1 μM, respectively (Figure 2b). These results were similar to the findings reported by Ying *et al.* [22].

### 3.3. Piperine Suppressed AA Liberation by Attenuating cPLA_2_ Activity in Platelets, but Not in Macrophages

**Figure 3:**
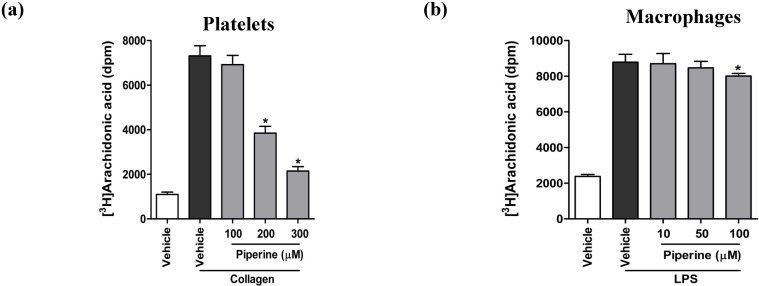
Piperine suppressed the liberation of the arachidonic acid in collagen-stimulated platelets, but not in LPS-stimulated RAW264.7 cells. [^3^H]AA-labeled platelets and RAW264.7 cells were treated with a range of concentrations of piperine (gray bars) or vehicle (DMSO, white and black bars), as presented in the graphs. The liberation of [^3^H]AA in platelets stimulated by 10 μg/mL collagen (**a**) and in RAW264.7 cells stimulated by 1 μg/mL LPS (**b**) was determined by measuring the radioactivity. Data are expressed as means ± S.D. of three separated experiments. * *p* < 0.05 *vs.* vehicle controls (black bars).

**Figure 4:**
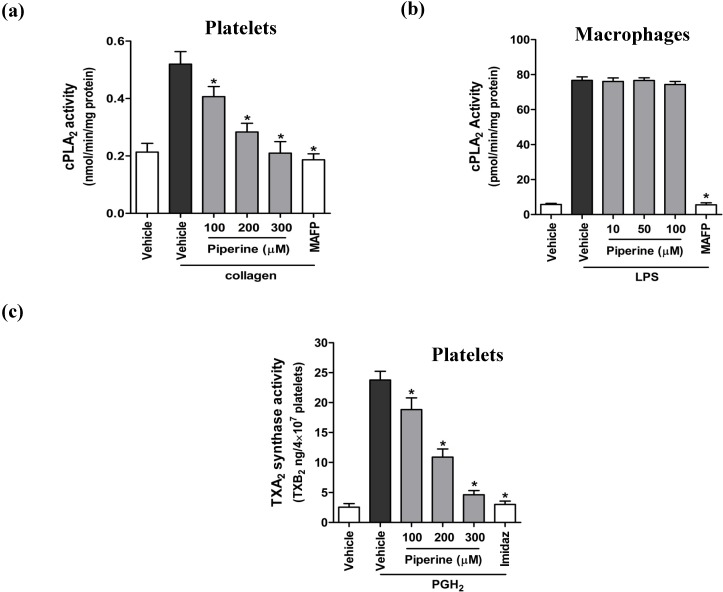
Piperine inhibited the activities of cPLA_2_ and TXA_2_ synthase in platelets, but not RAW264.7 cells. Platelets and RAW264.7 cells were treated with a range of concentrations of piperine (gray bars), vehicle (DMSO, white and black bars), or 10 μM MAFP (a cPLA_2_ inhibitor), as presented in the graphs. cPLA_2_ activity in platelets stimulated by 10 μg/mL collagen (**a**) and RAW264.7 cells stimulated by 1 μg/mL LPS (**b**) was measured as described in the Materials and Methods section. TXA_2_ synthase activity was quantified by measuring the production of TXB_2_; (**c**) TXA_2_ synthase activity in intact platelets was measured in the presence of piperine or 50 mM imidazole (a TXA_2_ synthase inhibitor). Data are expressed as means ± S.D. (*n* = 3). * *p* < 0.05 *vs.* vehicle controls (black bars).

To investigate the possible mechanisms of suppression of platelet aggregation and anti-inflammatory activities, we tested the effect of piperine on AA liberation in collagen-stimulated platelets and LPS-stimulated RAW264.7 cells. In fully aggregated, collagen-stimulated platelets, AA liberation increased significantly by 6.7-fold, relative to the unstimulated control cells. Treatment of platelets with the same piperine concentrations (100 to 300 μM) used in the platelet aggregation assay significantly suppressed collagen-induced AA liberation in a concentration-dependent manner (Figure 3a). In contrast, treatment of RAW264.7 cells with 10 to 100 μM piperine, which showed a significant inhibition of prostaglandin generation (Figure 2c,d), did not affect LPS-induced AA liberation (Figure 3b). These results suggest that piperine may affect platelet aggregation and inflammation through different mechanisms of action. Since piperine was observed to suppress AA liberation in collagen-stimulated platelets, we further tested the effect of piperine on the activity of cPLA_2_, an enzyme that catalyzes the release of AA from the platelet membrane phospholipids [36,37]. We found that piperine significantly inhibited collagen-induced cPLA_2_ activity in a concentration-dependent manner (Figure 4a), while eliciting no effect on LPS-stimulated cPLA_2_ activity in RAW264.7 cells (Figure 4b). We further confirmed that, under our experimental condition, cPLA_2_ inhibitor MAFP [38] significantly suppressed cPLA_2_ activity in both platelets and RAW264.7 cells (Figure 4a,b). Taken together, these findings suggest that piperine attenuates cPLA_2_ activity and suppresses AA liberation during platelet aggregation, but not in macrophage inflammation.

### 3.4. Piperine Inhibited Enzyme Activities of TXA_2_ Synthase and COX-2, but Not COX-1

**Figure 5:**
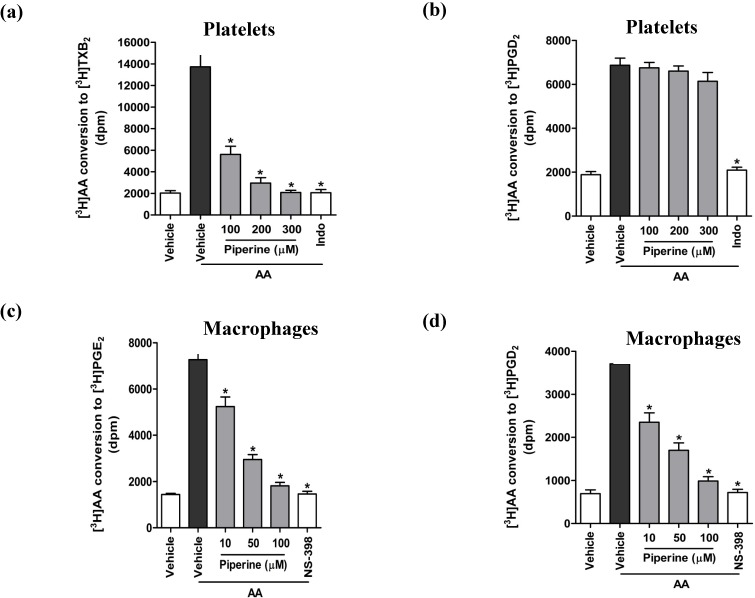
Piperine inhibited the activity of COX-2, but not COX-1. The conversion of exogenous AA to thromboxane and prostaglandins were measured as a reflection of COX activity. Platelets and RAW264.7 cells were treated with a range of concentrations of piperine, DMSO (vehicle), 50 μM indomethacin (a COX-1 inhibitor), or 1 μM NS-398 (a COX-2 inhibitor), as presented in the graphs. Platelets and RAW264.7 cells were subsequently incubated with a mixture of [^3^H]AA and the unlabeled AA for 10 min. The radioactivity corresponding to [^3^H]TXB_2_ (**a**) and [^3^H]PGD_2_ (**b**) measured in platelets was an index of COX-1 activity, while [^3^H]PGE_2_ (**c**) and [^3^H]PGD_2_ (**d**) signal measured in RAW264.7 cells corresponded to COX-2 activity. Data are expressed as means ± S.D. (*n* = 3). * *p* < 0.05 *vs.* vehicle controls (black bars).

Although previous studies have demonstrated that piperine inhibits inflammation through a suppression of COX-2 gene transcription and protein expression [16,20,22], the effect of piperine on the activity of other COX enzymes, particularly the COX-1 isozyme, remains unexplored. To address this knowledge gap, we evaluated the effect of piperine on COX activity by measuring the conversion of exogenous AA into metabolites such as TXB_2_ and prostaglandins, as an index of COX-1 and COX-2 activities. We found that the conversion of exogenous AA into TXB_2_ in platelets was significantly inhibited by piperine (Figure 5a), while the conversion of AA to PGD_2_ was not affected (Figure 5b). Both conversions were inhibited by indomethacin, a potent COX-1 inhibitor. Interestingly, we further found that TXA_2_ synthase activity was significantly inhibited by piperine (Figure 4c). These results indicate that piperine inhibits the generation of TXB_2_ from released AA in the platelets by the suppression of TXA_2_ synthase activity, but not through a direct suppression of COX-1 activity. In contrast, treatment of RAW264.7 cells with piperine significantly inhibited the conversion of AA into prostaglandins PGE_2_ (Figure 5c) and PGD_2_ (Figure 5d) in a concentration-dependent manner. Taken together, these findings demonstrate that piperine inhibits the generation of eicosanoids by suppressing the activities of COX-2 and TXA_2_ synthase, but does not affect COX-1 activity.

## 4. Discussion

Collectively, our results demonstrate that piperine is a bioactive alkaloid compound that exhibits activity against platelet aggregation and the macrophage inflammatory response through the regulation of the AA-metabolizing enzymes. Additionally, we demonstrated that piperine suppresses the enzyme activity of cPLA_2_ and TXA_2_, but not COX-1, in collagen-stimulated platelets. Conversely, piperine was shown to suppress the activity of COX-2, but not cPLA_2_, in LPS-stimulated RAW264.7 macrophage cell line.

Piperine is an alkaloid from the *Piper* species that has been reported to inhibit platelet aggregation. However, the mechanism of the anti-platelet aggregation action of piperine remains unknown. Piperine has been previously shown to inhibit the expression of COX-2, a key enzyme in the AA metabolic pathway, resulting in a decreased production of PGE_2_ in the inflammatory responses. We therefore investigated a possible anti-platelet mechanism of piperine through the modulation of the AA metabolic pathway. Moreover, we compared the effects of piperine on the liberation of AA and the activities of AA-metabolizing enzymes in collagen-stimulated platelets and LPS-stimulated RAW264.7 cells. In our study, piperine inhibited collagen- and AA-induced platelet aggregation but did not affect the response to U46619. These findings suggest that the anti-platelet activity of piperine may be mediated through the inhibition of collagen- and AA-stimulated platelet activation cascades, rather than through direct antagonism of the TXA_2_ receptor. Additionally, piperine was found to inhibit the generation of PGE_2_ and PGD_2_ in LPS-stimulated RAW264.7 cells. We also found that piperine has different ranges of effective concentration on platelet aggregation (100 to 300 μM) and macrophage inflammatory responses (10 to 100 μM), respectively. Comparing the anti-platelet aggregation and anti-inflammatory activities of piperine, our results showed that the IC_50_ values for the inhibition of platelet aggregation (ranging from 134.2 to 158.0 μM) were higher than those determined for the inhibition of macrophage inflammatory response (ranging from 7.7 to 10.1 μM). This suggests that the murine macrophages RAW264.7 cells are more sensitive to piperine than rabbit platelets, but the pharmacological basis of this difference is unclear. Previous studies have demonstrated that the active concentrations of piperine vary, depending on the cell type and animal species in which its effects are assessed [39,40]. Therefore, through further mechanism studies, we evaluated the activity of piperine at concentrations that exhibited inhibitory activity in each of the platelet aggregation assay and RAW264.7 prostaglandin-generation experiments.

AA, which is released from the membrane phospholipids by the action of PLA_2_, is a substrate for the generation of eicosanoids through further enzymatic metabolism. We demonstrated that the increased liberation of AA in collagen-stimulated platelets and stimulation of RAW264.7 cells by LPS was suppressed by piperine in platelets, but not in RAW264.7 cells. Furthermore, piperine significantly inhibited the collagen-induced activity of cPLA_2_ in platelets, with the inhibitory effect corresponding to the suppression of AA liberation and platelet aggregation. Conversely, piperine did not affect LPS-induced cPLA_2_ activity in macrophages. These results indicated that piperine may selectively inhibit cPLA2 activity depend on the stimulus response pathway. This suggests that piperine may affect collagen-induced platelet aggregation and LPS-stimulated macrophage inflammatory response through distinct mechanisms.

COX is the key enzyme required for the conversion of AA to eicosanoids, and two isoforms COX-1 and COX-2 are well known and characterized. COX-1 is constitutively expressed in most of tissues and functions in normal cell physiology, and the other inducible COX-2 is expressed in response to inflammatory stimuli such including LPS [41,42,43]. A recent study demonstrated that COX-1 expression and activity is not significantly regulated by LPS stimulation in macrophages, although basal level of COX-2 is expressed [44]. In contrast, the amount of COX-2 in platelets exists at very low amounts [45].This suggests that platelets and macrophages differentially express COX-1 or COX-2 in different physiological situations [46]. In our current study, we showed that piperine effectively inhibits the conversion of exogenous AA into PGE_2_ and PGD_2_ in RAW264.7 cells, which is a reflection of its effect on COX-2 activity. This finding is consistent with previous observations demonstrating that piperine decreases the production of pro-inflammatory mediators by inhibiting the COX-2 gene transcription and protein expression *in vitro* and *in vivo*. COX-1, a COX isozyme constitutively expressed in the platelets, mediates the synthesis of eicosanoids, including TXA_2_ and PGD_2_, responsible for platelet activation and aggregation. Since the effect of piperine on COX-1 has not been studied yet, we investigated the effect of piperine on the activity of this isozyme in platelets. We found that piperine significantly inhibited the conversion of exogenous AA into TXB_2_, but not PGD_2_, a metabolite formed by COX-1 activity. This result indicates that the inhibition of TXB_2_ generation from AA by piperine is not dependent on COX-1 activity.

TXA_2_ synthase is an important AA-metabolizing enzyme, which converts PGH_2_ into TXA_2_ following the generation of PGH_2_ by COX-1 from AA in the platelets [4,35]. Interestingly, we found that TXA_2_ synthase activity was significantly inhibited by piperine, indicating that piperine inhibits TXB_2_ generation from AA in platelets by the suppression of TXA_2_ synthase activity, rather than through the suppression of COX-1 activity. While our results do not rule out the possibility of the involvement of other piperine-targeted enzymes (e.g., PGE_2_ and PGD_2_ synthases), our findings clearly demonstrate that piperine inhibits the generation of eicosanoid mediators from AA by the suppression of TXA_2_ synthase in platelets and selective inhibition of COX-2 in macrophages.

## 5. Conclusions

**Figure 6:**
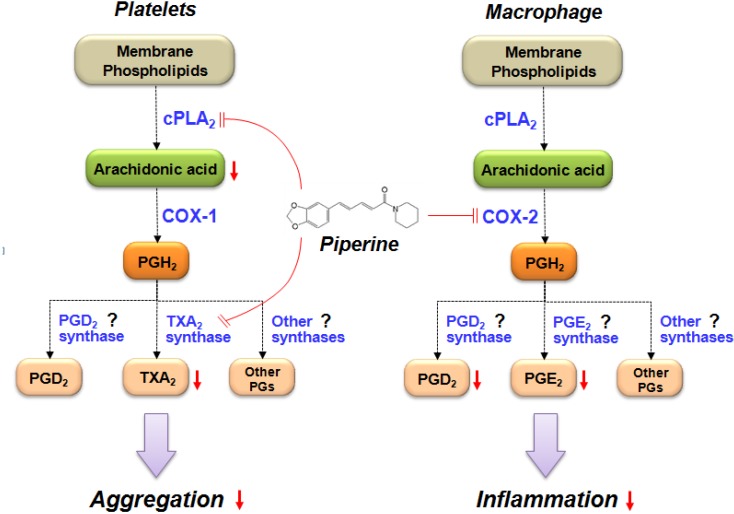
Scheme of the proposed mechanism by which piperine inhibits platelet aggregation and macrophage inflammatory processes. This study demonstrated that piperine (Figure 1a) inhibits both platelet aggregation (Figure 1b) and macrophage inflammatory responses (Figure 2). Treatment with piperine was shown to suppress AA liberation (Figure 3a) through the inhibition of cPLA_2_ activity (Figure 4a) in platelets, but not in macrophages (Figure 3b and Figure 4b). Additionally, piperine inhibits the activities of TXA_2_ synthase (Figure 4c) and COX-2 (Figure 5c,d), but not COX-1 (Figure 5a,b). Piperine inhibits cPLA_2_ and TXA_2_ activities, suppressing platelet aggregation through decreased TXA_2_ generation. Piperine-dependent inhibition of COX-2 activity results in a suppression of the macrophage inflammatory response through decreased prostaglandin generation.

In conclusion, as summarized in Figure 6, piperine inhibits platelet aggregation by inhibiting cPLA_2_ and TXA_2_ activities but does not affect COX-1. In contrast, piperine inhibits macrophage inflammatory response by inhibiting COX-2 activity without effects on cPLA_2_. This demonstrates that piperine inhibits both platelet aggregation and macrophage inflammatory response via regulation of the AA-metabolizing pathways, but by different inhibitory effects on the metabolic enzymes. Further studies are warranted to determine the exact mechanism of inhibition of the activities of AA-metabolizing enzymes of piperine, and to investigate how it can be used in anti-thrombotic and anti-inflammatory therapies.
